# Clozapine-Induced Obsessive-Compulsive Symptoms in Schizophrenia: A Critical Review

**DOI:** 10.2174/157015912799362724

**Published:** 2012-03

**Authors:** Frederike Schirmbeck, Mathias Zink

**Affiliations:** Central Institute of Mental Health, Department of Psychiatry and Psychotherapy, P.O. Box 12 21 20, D-68072 Mannheim, Germany

**Keywords:** Clozapine, comorbidity, compulsive, obsessive, pharmacology, schizophrenia, serotonin.

## Abstract

Obsessive-compulsive disorder (OCD) is rarely associated with schizophrenia, whereas 20 to 30% of schizophrenic patients, suffer from comorbid obsessive-compulsive symptoms (OCS). So far no single pathogenetic theory convincingly explained this fact suggesting heterogeneous subgroups. Based on long-term case observations, one hypothesis assumes that second-onset OCS in the course of schizophrenia might be a side effect of second generation antipsychotics (SGA), most importantly clozapine (CLZ). This review summarizes the supporting epidemiological and pharmacological evidence: Estimations on prevalence of OCS increase in more recent cross-sectional studies and in later disease stages. Longitudinal observations report the *de novo*-onset of OCS under clozapine treatment. This association has not been reported with first generation antipsychotics (FGA) or SGAs with mainly dopaminergic mode of action. Finally, significant correlations of OCS-severity with duration of treatment, dose and serum levels suggest clozapine-induced OCS. However, supposed causal interactions need further verifications. It is also unclear, which neurobiological mechanisms might underlie the pathogenetic process. Detailed genotypic and phenotypic characterizations of schizophrenics with comorbid OCS regarding neurocognitive functioning and activation in sensitive tasks of functional magnetic imaging are needed. Multimodal large-scaled prospective studies are necessary to define patients at risk for second-onset OCS and to improve early detection and therapeutic interventions.

## OBSESSIVE-COMPULSIVE SYMPTOMS IN SCHIZOPHRENIA

Schizophrenic patients have a high lifetime risk for comorbid obsessive-compulsive symptoms (OCS). Several epidemiological studies report that 20 to 30% suffer from obsessive, distressing, intrusive thoughts and related compulsions (e.g. repeated hand washing, checking behaviour, counting or cleaning) conceived as attempts to neutralize the obsessions [1-5]. A large proportion of schizophrenics with OCS fulfil current diagnostic criteria of obsessive-compulsive disorder (OCD). In contrast, primary OCD-patients most frequently present comorbid affective or anxiety disorders and only 1.7% suffer from comorbid psychotic symptoms [6]. Comorbid OCS in schizophrenia is associated with pronounced positive and negative symptoms [7], lower levels of social functioning, higher treatment costs, worse social and vocational rehabilitation. Consequently they have a less favourable prognosis [8-11], in particular if recent concepts of response, remission and recovery [12,13] are applied. These well-documented facts correct former concepts, where comorbid OCS in schizophrenia were assumed to have protective effects regarding psychotic desintegration [14-18]. It is worth noting, that somatic obsessions and hoarding might indeed compensate psychotic anxiety and disorganization [11]. 

## PATHOGENETIC THEORIES

The considerably high coincidence of OCS and schizophrenia deserves a neurobiological explanation, but so far the pathogenetic processes of this comorbidity could not be clearly unravelled.

Several heterogeneous theories have been proposed: A small minority of patients might coincidently suffer from both schizophrenia and OCS, representing random associations of two common disorders. 

Within the spectrum of OCD, the concept of “schizotypic OCD” has been described [19,20] integrating the assumption that primary OCD-patients present cognitions that migrate on a spectrum between obsessions and delusions. A similar concept of “obsessions without insight” was integrated into current diagnostic systems making the differentiation between obsessions and delusions more difficult. OCD-patients without insight might represent a subgroup with genetic, phenotypic and therapeutic vicinity to the schizophrenia-spectrum [21,22]. Using stepwise regression models, Guillem *et al*. showed positive correlations between delusions and obsessions, as well as hallucinations and compulsions suggesting common pathogenetic mechanisms [11]. 

Within the spectrum of schizophrenia, a so-called “schizo-obsessive” subtype of psychosis has been proposed [23-27]; specific subtypes of OCS were perceived as part of the basic symptom cluster in the early course of schizophrenia [28,29].

Furthermore, catatonic symptoms of schizophrenia overlap with the obsessive-compulsive phenotype [30]. This circumstance limits the precision of psychometric scales such as the catatonia rating scale (CRS) [31] and the Yale-Brown-Obsessive-Compulsive Scale (YBOCS) [32,33]. However, descriptions of the natural long-term course of schizophrenia, for instance published by Karl Leonhard [34], allow clear discrimination between OCS and catatonic symptoms most importantly in patients with so-called “manierierte Katatonie” and do not support the view that OCS might be a part of the residual state.

The summarised pathogenetic concepts are matters of current discussion. They strongly suggest a dimensional perspective on OCS and psychosis with heterogeneous subgroups. Progress in pathogenetic understanding and therapeutic implications will be achieved if differential phenotypic correlates can be defined for homogeneous samples. A remarkably simple clinical assessment of three important events allows a rough, but useful categorization of comorbid patients: 1. When did the first psychotic manifestation occur? 2. When was antipsychotic treatment initiated? 3. When did OCS develop or showed – if pre-existing – a marked aggravation? 

## SECOND-ONSET OCS INDUCED BY ANTISEROTONERGIC ANTIPSYCHOTICS

Applying the above mentioned characterization by clinical events, a subgroup of comorbid patients is defined by the order 1 – 2 – 3: These patients experienced the *de novo*-onset of OCS or a marked aggravation of OCS severity after treatment initiation with second generation antipsychotics (SGA), most importantly clozapine (CLZ). Noteworthy, SGA carry the important pharmacodynamic feature of balanced antidopaminergic and antiserotonergic properties that markedly exceed 5HT-receptor blockade by first generation antipsychotics (FGA) [35,36]. Starting with the observations of Baker *et al. *[37] and De Haan *et al*. [38] the hypothesis of SGA-induced OCS first came up [39]. Since then several studies support this assumption, especially for CLZ [40-42]. 

Nevertheless, CLZ must be considered a necessary and indispensable part of the antipsychotic armament [43,44]. As early as 1988, Kane *et al.* provided first evidence that CLZ might improve treatment resistant psychoses [45]. Today its antipsychotic efficacy has been demonstrated to be superior in the treatment of refractory schizophrenia, in independent investigations [46], including the CATIE-study [47]. Therefore, CLZ is the antipsychotic of first choice in treatment resistant schizophrenia. In addition, the substance embarks important protective effects against suicidal behaviour resulting in lowest mortality of schizophrenics as documented in the large, naturalistic FIN11-study [48]. However, the *de novo* occurrence or exacerbation of OCS has most often been observed during treatment with CLZ [39,41] and several epidemiological and pharmacological arguments support the attribution of pro-obsessive effects.This review aims at summarizing the evidence for OCS-induction by CLZ in order to provide a basis for pathogenetic considerations and the design of further clinical trials. For this task, we systematically screened medical databases (Medline OVID, PSYNDEX and PsycINFO) for publications until June 2011 using the keywords (“schizophrenia” or “Psychosis” or “psychotic disorder”) linked with (“OCD” or “obsessive” or “compulsive”). Within these results, we selected publications that address the question of SGA- and more specifically clozapine-induced OCS in schizophrenia. Studies were categorized according to epidemiological (see Table **1**) and pharmacological (see Table **2**) arguments.

## EPIDEMIOLOGY

Epidemiological studies on OCS in schizophrenia (Table **1**) differed markedly in sample properties, applied psychometric procedures and diagnostic criteria. Furthermore, a potential publication bias and changes of general awareness over time have to be considered. Nevertheless, several conclusions can be drawn: 

### Increase of OCS Prevalence after Market Approval of SGAs

The interrelations between psychotic disorders and OCS were first described by Westphal [17], but not before the last decades of the 20^th^ century notable concern about this problem arose. As summarized by Mukhopadhaya *et al*. [3], estimations on prevalence of OCS in schizophrenia vary between 0.5 and 55% [3]. Only a minority of investigations report high comorbidity rates in samples under FGA treatment [49-52], but the awareness of OCS in schizophrenia increased with the market introduction of CLZ in the 1970ies in Europe and the late 1980ties in the USA [43,53]. CLZ differed from FGAs due to pharmacodynamic properties as a potent serotonergic antagonist [35,36]. Simultaneously estimations about prevalence of comorbid OCS rose up to 30% [2-5]. 

### Increase of OCS Prevalence after First Onset of Psychosis

Prevalence estimations of OCS in samples at ultra high risk for psychosis [54,55] or in first episode patients (FEP) [56] are considerably lower. Reported comorbidity rates range from 7% in a sample of 200 FEPs [42] over 9.3% in 193 [57] up to 14% in 50 FEPs [56]. In a study by Shioiri *et al*. only 3 of 219 patients were diagnosed with OCD at onset of psychosis [58] and within 121 recent onset psychotic disorders only 1.3% showed OCS under treatment with antipsychotics excluding CLZ [38]. These prevalence rates in early stages of the disease markedly contrast with high comorbidity rates in cross-sectional studies of mixed disease stages [2-5], suggesting that a significant proportion develops OCS during or even as a consequence of anti-psychotic treatment. 

### Onset of *de novo* OCS during Antipsychotic Treatment

Several case reports [59,60], cases series [61] and systematic evaluations [38,39,42] describe the de-novo emergence of OCS during the treatment with atypical antipsychotics. As described in Table **1**, De Haan *et al.* reported OCS development within several months after treatment initiation with CLZ in 20.6% of recent-onset patients [38]. Poyurovski *et al*. estimated that up to 70% of schizophrenics treated with antiserotonergic SGAs such as CLZ, olanzapine or risperidone develop secondary OCS [1]. Independent studies report equally high numbers of SGA-induced OCS within samples of comorbid patients: 25 of 28 [62], 29 of 39 [63] and 23 of 26 [64].

Extending the perspective from epidemiology to pharmacology, further arguments have to be considered (see Table **2** for summary).

## PHARMACOLOGICAL EVIDENCE:

### Higher Prevalence of OCS in Patients Treated with CLZ

The risk of suffering from OCS markedly differs if patients are stratified according to their mode of antipsychotic treatment. High prevalence in groups treated with CLZ markedly contrast with lower prevalence and severity of OCS for example in patients treated with the FGA haloperidol (HAL) [7]. Ertugrul *et al*. reported a prevalence of 76% for OCS in clozapine-treated patients and based on retrospective assessment de novo onset or exacerbation in 38% of them [65]. Within SGAs, marked differences exist in specific pharmacodynamic properties, in particular regarding inherent serotonergic blockade, monoaminergic reuptake inhibition or even partial serotonergic agonism [66-70]. The partial dopaminergic and serotonergic agonist aripiprazole *per se* was associated with an inherent anti-obsessive potency [59,61,71-73], quite similar to amisulpride, a substance with nearly exclusive affinity to dopamine D3/D2 receptors [74,75]. Our workgroup conducted a cross-sectional analysis of 70 schizophrenic patients under monotherapy with CLZ or olanzapine (group I) vv amisulpride or aripiprazole (group II). Results showed that 71.8% of group-I-patients suffered from OCS while only 9.7% of patients in group-II reported OCS. In cohort I, 16 of 39 investigated patients (41%) reported YBOCS scores above 16 representing clinically meaningful OCS [62]. *Vice versa*, a stratification of schizophrenics according to presence or absence of comorbid OCS revealed CLZ treatment in 76.9% versus 35.9% [64]. These results strongly suggest associations between CLZ treatment and OCS development. However, confounding effects due to the selection of specific SGAs for specific subgroups need to be considered.

## INFLUENCE OF DURATION OF TREATMENT WITH ANTISEROTONERGIC SGAS ON OCS

Lin *et al*. compared CLZ-treated patients with and without comorbid OCS and reported no difference in duration of illness, but significantly longer CLZ treatment [63]. Similarly, a positive correlation of OCS severity, with duration of treatment, was found for the subgroup of CLZ treated schizophrenics in our sample mentioned above [62]. Parallel observations were reported by De Haan *et al.* regarding the closely related SGA olanzapine: Severity of OC symptoms significantly correlated with duration of olanzapine treatment [76]. 

## INFLUENCE OF TREATMENT DOSE AND BLOOD SERUM LEVELS OF CLZ ON OCS SEVERITY

In addition to the association with duration of treatment, a positive correlation between dose or serum levels of CLZ and severity of OCS has been reported in independent samples [3,41,63]. In line with these previous results, we found positive correlations between the daily dose of CLZ and OCS severity [62].

## REDUCTION OF OCS SEVERITY AFTER DOSE REDUCTION

OCS during treatment with CLZ often markedly improve after dose reduction, for instance due to combinations with other SGAs such as aripiprazole [59,61,77]. This might be an indirect hint towards a suggested dose-related side effect of CLZ. However, because aripiprazole itself exerts anti-obsessive effects due to its partial dopaminergic and serotonergic agonism, evidence from combination trials is limited. 

In summary, comorbid OCS in schizophrenia is clearly associated with the antiserotonergic SGA CLZ. Pharmacological arguments based on correlations between OCS severity and dosage as well as duration of CLZ application indicate a causal interaction and suggest OCS induction as a side-effect. The SGA CLZ may therefore carry pharmacodynamic features that justify the characterization as a ‘switch from psychotic to obsessive’. Specific individual conditions, such as the subtype of schizophrenia, the stage of the illness, any affective comorbidity, and a family history for anxiety disorders might modify the liability to develop OCS during CLZ treatment.

However, conflicting results exist: Several authors reported OCS reduction in schizophrenia after the addition of CLZ [78] or after an increase in dosage [39]. These diverging findings might be due to the mentioned diagnostic difficulties in differentiating between OCS and delusional or catatonic symptoms of schizophrenia and the heterogeneity within comorbid clinical samples. 

For patients with primary OCD, exhibiting treatment-resistance to serotonergic antidepressants, favourable effects of SGAs, including those with antiserotonergic properties such as risperidone, have been reported [79-81]. However, even here, current treatment guidelines do not recommend CLZ as an augmentation for treatment-resistant OCD. Standard research strategies to evaluate causal interactions are *placebo*-controlled, randomized, prospective trials. However, for ethical and legal reasons, this design can not be applied to interventions involving CLZ. Therefore, psychiatric research in this field is faced with a vicious circle that cannot be overcome by merely cross-sectional perspectives: OCS comorbidity is associated with CLZ treatment - CLZ is the SGA of choice for treatment resistant schizophrenia, the most severely affected patients - severe psychotic positive and negative symptoms are associated with OCS – OCS comorbidity is associated with CLZ. Only prospective, multidimensional investigations in sufficiently homogeneous samples can help to differentiate between causes and consequences. 

## MECHANISM OF ACTION

Within the current pathogenetic theories of obsessions and compulsions, a dysregulation of serotonergic neurotransmission in a network comprising cortical, striatal and thalamic centres has been proposed [82]. It is assumed that CLZ induces OCS due to its strong inherent antiserotonergic properties [43,44,83], most importantly the antagonism at 5-HT1C, 5-HT2A and 5HT2C receptors [69,84,85]. This theory is in line with therapeutic effects of SSRIs (serotonin specific reuptake inhibitors) and changes of serotonergic neurotransmission after successful cognitive behavioural therapy (CBT) in OCD [86,87]. Reciprocal interactions of antipsychotics with dopaminergic and serotonergic receptors leading to altered glutamatergic neurotransmission must also be considered [68]. In addition to the pharmacodynamic mechanism, it might be discussed whether specific genetic properties dispose schizophrenic patients to develop secondary OCS during treatment with SGAs. One candidate polymorphism has been located in the gene *SLC1A1 *(former nomenclature EAAC1: excitatory amino acid carrier 1) encoding the neuronal glutamate transporter which has been independently associated with a genetic risk for OCD [88-90]. In a genetic association study with this candidate gene, Kwon *et al*. [40] reported significant associations of specific SNPs (single nucleotide polymorphisms) with the development of OCS during treatment with SGAs, but a replication approach in a Caucasian sample was unable to confirm these results [91].

## NEUROCOGNITIVE CHARACTERIZATION OF COMORBID PATIENTS

Neurocognitive comparisons between schizophrenics with and without OCS revealed several qualitative and quantitative domain specific differences, but detailed characterization of patients with SGA-induced OCS is still needed. In general pronounced cognitive deficits in OCS patients have been reported for domains of visual memory and executive functioning [4,92-96]. Accordingly, we found marked impairment in our CLZ-treated OCS-positive patients in visual memory, impulse inhibition, perseveration and set-shift abilities [62]. These cognitive deficits correlated with OCS severity and might therefore be linked to the pathomechanism of OCS in schizophrenia. In the future, neurocognitive assessment prior or during antipsychotic treatment might help to define patients at risk for secondary OCS and provide important information for clinical decisions. The detection of OCS during early stages of treatment, is particularly important, since recent studies showed that up to 50% of patients suffering from comorbid OCS were previously undiagnosed during routine psychiatric treatment [3]. 

## CONCLUSIONS

Comorbid OCS in schizophrenia are a common clinical problem. For a subgroup of these patients, several lines of evidence strongly suggest an induction of second-onset OCS during CLZ treatment. For legal and ethical reasons, this hypothesis cannot be addressed in randomized controlled trials of first-episode patients. We therefore suggest longitudinal follow-up investigations of groups defined by their psychopharmacological treatment in a head-to-head design and comparisons of within-group changes of OCS over time. These approaches will be able to corroborate the hypothesis of CLZ-induced OCS and pin down prodromal and early signs of comorbid OCS through neurocognitive characterisations and functional neuroimaging. In terms of therapeutic implications several approaches have been suggested [97]. Regarding pharmacological interventions preliminary evidence shows positive effects of CLZ dose reduction after augmentation with mood stabilizers [60,98] or in combination with anti-obsessive SGAs [59,61,77]. However no treatment guidelines exist so far and clinical trials on cognitive, behavioural therapy are completely missing.

## Figures and Tables

**Table 1:** Epidemiological Evidence

Argument		References	Number of Patients and Clinical Characterization	Design	Main Findings
**Increase of prevalence from prodromal states over first episode samples to chronic course of schizophrenia**	**At risk mental state samples**	Rubino *et al*. 2009 [55]	197 Schizophrenic patients	Retrospective assessment of morbidity before the age of 18	8 % of schizophrenic patients suffer from OCD before age of 18
		Niendam *et al*. 2009 [54]	64 UHR-patients	Cross-sectional survey using SIPS and Padua-inventory	20 % of UHR patients report OCS. This comorbid subgroup, however, shows a lower risk for conversion into psychosis.
	**First episode patients**	De Haan *et al*. 2004 [42]	196 FEP	Retrospective chart study	7 % of FEPs showed OCS at first manifestation.
		Shioiri *et al*. 2007 [58]	219 FEP	Retrospective assessment of OCS-prevalence	3 % of FEPs showed OCS in the prodromal phase and 1.5 % fulfil diagnostic criteria for OCD at first manifestation
		Poyurovsky *et al*. 1999 [56]	50 FEP	Cross-sectional assessment of OCD comorbidity	At first manifestation of psychosis, 14 % fulfil criteria for OCD.
		Sterk *et al*. 2011 [57]	194 FEP	Cross-sectional assessment of OCS comorbidity	At first manifestation of psychosis, 9.3 % fulfil criteria for OCD.
	**Schizophrenic patients**	Mukhopadhaya *et al*. 2009 [3]	1972	Review of studies reporting on OCS prevalence in schizophrenia	High variability. Mean prevalence of 22 % reviewing data on 1972 patients.
		Buckley *et al*. 2009 [2]	3656	Review of studies reporting on OCS prevalence in schizophrenia	Mean prevalence of 23 %.
		Lysaker *et al*. 2009 [4]	Not specified	Review of studies reporting on OCS prevalence in schizophrenia	Amongst schizophrenic patients, more than one third suffers from clinically significant OCS, 10 to 25 % meet diagnostic criteria of OCD
***De novo * onset or exacerbation of OCS during antipsychotic treatment**		Case reports Zink *et al*. 2006 and 2007 [59,60] and Case series Englisch *et al*. 2009 [61]		Longitudinal observation of course of illness	First manifestation and start of antipsychotic treatment precede onset of OCS
		De Haan *et al*. 1999 [38]	121 recent-onset schizophrenic patients	Longitudinal observation of course of illness	Emergence or increase of OCS in 1.3 % of non-clozapine treated and 20.6 % of clozapine-treated patients
		Lykouras *et al*. 2003 [39]	55 schizophrenia patients	Systematic review of published case reports	Until 2003, a *de novo *onset or exacerbation of OCS had been published regarding clozapine (N=30), risperidone (N=16), olanzapine (N=8) and quetiapine (N=1)
		De Haan *et al.* 2004 [42]	200 recent-onset schizophrenic patients	Longitudinal observation of course of illness	Emergence or increase of OCS in 0 % of non-clozapine treated and 9.8 % of clozapine-treated patients.
**Proportion of SGA-induced OCS within the complete comorbid sample**		Lin *et al.* [63]	CLZ: 102	Cross-sectional: Stratification for CLZ-treatment with or without OCS	Within 39 clozapine-treated patients with OCS, 29 were classified as clozapine-induced.
		Lim *et al*. 2007 [64]	Total sample: 209, comorbid subsample: 26	Cross-sectional. Stratification for SZ with or without OCS	Within 26 schizophrenics with SGA-associated OCS, only 3 had a history of transient OCS before the onset of psychosis
		Schirmbeck *et al*. 2011 [62]	CLZ: 26 OLZ: 13	Cross-sectional. Stratification for treatment with SGAs in monotherapy	Within 39 patients, 28 showed OCS, but only 3 reported OCS before or at onset of psychosis.

Epidemiological evidence in favour of an increase of OCS-prevalence during course of illness suggests the *de novo*-onset of an SGA-induced side effect. Abbreviations: FEP: first episode schizophrenic patients; FGA: first generation antipsychotics; OCD: obsessive compulsive disorder; OCS: obsessive compulsive symptoms; OLZ: olanzapine; SGA: second generation antipsychotics; SIPS: Structured interview for prodromal symptoms; SZ: schizophrenia and schizophrenia spectrum disorders; UHR: Ultra high risk

**Table 2.** Pharmacological Evidence

Argument	Reference	Number of Patients	Design	Main Findings
**Association of CLZ with comorbid OCS**	Lim *et al*. 2007 [64]	Total sample: 209, comorbid subsample: 26	Cross-sectional. Stratification for SZ with or without OCS	CLZ-treatment in 35.9 % of the total sample, but in 76.9 % of the comorbid patients
**Association of OCS with OLZ or CLZ**	Sa *et al*. 2009 [7]	CLZ: 40 HAL: 20	Cross-sectional. Stratification for treatment with CLZ or HAL	Prevalence of OCS 20 % (CLZ) vs. 10 % (HAL). Higher severity of OCS with CLZ
	Ertugrul *et al*. 2005 [65]	CLZ: 50	Cross-sectional. Stratification of treatment with CLZ	Within 50 patients treated with CLZ, 76% showed OCS. 20 % reported retrospectively *de novo* onset and 18 % an exacerbation.
	Schirmbeck *et al*. 2011 [62]	CLZ: 26 OLZ: 13 AMS: 15 APZ:16	Cross-sectional. Stratification for treatment with SGAs in monotherapy	Prevalence of OCS 71.8 % in CLZ or OLZ vs. 9.7 % in AMS or APZ. Highest severity of OCS with CLZ
**Correlation of OCS with duration of treatment**	Lin *et al.* [63]	CLZ: 102	Cross-sectional: Stratification for CLZ-treatment with or without OCS	Duration of CLZ-treatment significantly longer in CLZ-OCS-patients (82 vs. 56 months), no difference in duration of illness
	Schirmbeck *et al*. [62]	CLZ: 26	Cross-sectional: Stratification for CLZ-monotherapy	Duration of CLZ-treatment correlates positively with OCS severity (YBOCS, R=0.59).
**Correlation of OCS with CLZ-dosage or plasma concentration**	Reznik *et al*. [41]	N=15	Cross-sectional: Stratification for CLZ-therapy	Dosage-related, pro-obsessive influence of CLZ
	Mukhopadhaya *et al*. 2009 [3]	N=59	Cross-sectional: Stratification for CLZ-therapy	Higher CLZ-dosage in patients with comorbid OCS (432 mg/day) than without (351 mg/day)
	Schirmbeck *et al*. [62]	CLZ: 26	Cross-sectional: Stratification for CLZ-monotherapy	CLZ-dosage correlates positively with OCS severity (YBOCS, R=0.50).
	Lin *et al.* [63]	CLZ: 102	Cross-sectional: Stratification for CLZ-treatment with or without OCS	Higher plasma concentrations in CLZ-treated patients with OCS (595 ng/L) than without OCS (434 ng/L).
**Improvement after CLZ dose-reduction**	Rocha *et al.* 2006 [77]	3	Longitudinal observation of OCS severity	Reduction of OCS severity after CLZ down-tapering in combination with APZ
	Zink *et al.* 2006 [59]	1	Longitudinal observation of OCS severity	Reduction of OCS severity from YBOCS 24 to 19 after reduction of CLZ from 500 to 250 mg/die and combination with APZ (30 mg)
	Englisch *et al*. 2009 [61]	7	Longitudinal observation of OCS severity	Reduction of OCS severity from YBOCS 19 to 12 after reduction of CLZ from 364 to 293 mg/die and combination with APZ (23 mg)

Pharmacological evidence in favour of an association between clozapine-treatment and OCS. Abbreviations: AMS: amisulpride; APZ: aripiprazole; CLZ: clozapine; FGA: first generation antipsychotics; HAL: haloperidol; OCS: obsessive compulsive symptoms; OLZ: olanzapine; SGA: second generation antipsychotics; SZ: schizophrenia and schizophrenia spectrum disorders; YBOCS: Yale-Brown-Obsessive-Compulsive Scale.

## References

[1] Poyurovsky M, Weizman A, Weizman R (2004). Obsessive-compulsive disorder in schizophrenia: clinical characteristics and treatment. CNS Drugs.

[2] Buckley PF, Miller BJ, Lehrer DS, Castle DJ (2009). Psychiatric comorbidities and schizophrenia. Schizophr. Bull.

[3] Mukhopadhaya K, Krishnaiah R, Taye T, Nigam A, Bailey AJ, Sivakumaran T, Fineberg NA (2009). Obsessive-compulsive disorder in UK clozapine-treated schizophrenia and schizoaffective disorder: a cause for clinical concern. J. Psychopharmacol.

[4] Lysaker PH, Whitney KA (2009). Obsessive-compulsive symptoms in schizophrenia: prevalence, correlates and treatment. Expert Rev. Neurother.

[5] Achim AM, Maziade M, Raymond E, Olivier D, Merette C, Roy MA (2011). How prevalent are anxiety disorders in Schizophrenia? A meta-analysis and critical review on a significant association. Schizophr. Bull.

[6] de Haan L, Dudek-Hodge C, Verhoeven Y, Denys D (2009). Prevalence of psychotic disorders in patients with obsessive-compulsive disorder. CNS Spectrums.

[7] Sa AR, Hounie AG, Sampaio AS, Arrais J, Miguel EC, Elkis H (2009). Obsessive-compulsive symptoms and disorder in patients with schizophrenia treated with clozapine or haloperidol. Compr. Psychiatry.

[8] Cunill R, Castells X, Simeon D (2009). Relationships between obsessive-compulsive symptomatology and severity of psychosis in schizophrenia: a systematic review and meta-analysis. J. Clin. Psychiatry.

[9] Lysaker PH, Lancaster RS, Nees MA, Davis LW (2004). Patterns of obsessive-compulsive symptoms and social function in schizophrenia. Psychiatry Res.

[10] Öngür D, Goff DC (2005). Obsessive-compulsive symptoms in schizophrenia: associated clinical features, cognitive function and medication status. Schizophr. Res.

[11] Guillem F, Satterthwaite J, Pampoulova T, Stip E (2009). Relationship between psychotic and obsessive compulsive symptoms in schizophrenia. Schizophr. Res.

[12] Andreasen NC, Carpenter WT, Kane JM, Lasser RA, Marder SR, Weinberger DR (2005). Remission in Schizophrenia: proposed criteria and rationale for consensus. Am. J. Psychiatry.

[13] Jaeger M, Messer T, Laux G, Pfeiffer H, Naber D, Schmidt LG, Gaebel W, Klosterkötter J, Heuse I, Maier W, Lemke MR, Ruether E, Buckkremer G, Gastpar M, Riedel M, Bottlander R, Strauss A, Moeller H-J (2008). Standardized remission criteria in schizophrenia: descriptive validity and comparability with previously used outcome measures. Pharmacopsychiatry.

[14] Rosen I (1957). The clinical significance of obsessions in schizophrenia. J. Mental Sci.

[15] Dowling FG, Pato MT, Pato CN (1995). Comorbidity of obsessive-compulsive and psychotic symptoms: a review. Harvard Rev. Psychiatry.

[16] Stengel E (1945). A study on some clinical aspects of the relationship between obsessional neurosis and psychotic reaction types. J. Mental Sci.

[17] Westphal K, Ueber Z (1878). Archiv für Psychiatrie und Nervenkrankheiten.

[18] Zink M, Englisch S, Dressing H (2008). Neurobiology confirms Psychopathology: On the antagonism of psychosis and obsessive-compulsive syndromes. Psychopathology.

[19] Poyurovsky M, Faragian S, Pashinian A, Heidrach L, Fuchs C, Weizman R, Koran L (2008). Clinical characteristics of schizotypal-related obsessive-compulsive disorder. Psychiatry Res.

[20] Poyurovsky M, Koran LM (2005). Obsessive-compulsive disorder (OCD) with schizotypy vs. schizophrenia with OCD: diagnostic dilemmas and therapeutic implications. J. Psychiatric Res.

[21] Catapano F, Perris F, Fabrazzo M, Cioffi V, Giacco D, De S V, Maj M (2010). Obsessive-compulsive disorder with poor insight: a three-year prospective study. Prog. Neuro-Psychopharmacol. Biol. Psychiatry.

[22] Tumkaya S, Karadag F, Oguzhanoglu NK, Tekkanat C, Varma G, Ozdel O, Atesci F (2009). Schizophrenia with obsessive-compulsive disorder and obsessive-compulsive disorder with poor insight: a neuropsychological comparison. Psychiatry Res.

[23] Rajkumar RP, Reddy YC, Kandavel T (2008). Clinical profile of "schizo-obsessive" disorder: a comparative study. Compr. Psychiatry.

[24] Reznik I, Kotler M, Weizman A (2005). Obsessive and compulsive symptoms in schizophrenia patients--from neuropsychology to clinical typology and classification. [comment]. J. Neuropsychiatry Clin. Neurosci.

[25] Hwang MY, Morgan JE, Losconzcy MF (2000). Clinical and neuropsychological profiles of obsessive-compulsive schizophrenia: a pilot study.[see comment]. J. Neuropsychiatry Clin. Neurosci.

[26] Bottas A, Cooke RG, Richter MA (2005). Comorbidity and pathophysiology of obsessive-compulsive disorder in schizophrenia: is there evidence for a schizo-obsessive subtype of schizophrenia?. J. Psychiatry Neurosci.

[27] Sevincok L, Akoglu A, Arslantas H (2006). Schizo-obsessive and obsessive-compulsive disorder: comparison of clinical characteristics and neurological soft signs. Psychiatry Res.

[28] Ebel H, Gross G, Klosterkotter J, Huber G, Ebel H, Gross G, Klosterkotter J, Huber G (1989). Basic symptoms in schizophrenic and affective psychoses. Psychopathology.

[29] Sullwold L, Huber G (1986). Basic schizophrenic disorders. Monographien aus dem Gesamtgebiete der Psychiatrie:. Psychiatry Series.

[30] Fink M, Taylor MA (2001). The many varieties of catatonia. Eur. Arch. Psychiatry Clin. Neurosci.

[31] Bräunig P, Krüger S, Shugar G, Höffler J, Börner I (2000). The catatonia rating scale I--development, reliability, and use. Compr. Psychiatry.

[32] Woody SR, Steketee G, Chambless DL (1995). Reliability and validity of the Yale-Brown obsessive-compulsive scale. Behav.Res. Ther.

[33] de Haan L, Hoogeboom B, Beuk N, Wouters L, Dingemans PM, Linszen DH (2006). Reliability and validity of the Yale-Brown obsessive-compulsive scale in schizophrenia patients. Psychopharmacol. Bull.

[34] Beckmann H, Bartsch AJ, Neumärker K-J, Pfuhlmann B, Verdaguer MF, Franzek E (2000). Schizophrenias in the Wernicke-Kleist-Leonhard school. Am. J. Psychiatry.

[35] Meltzer HY (1995). Role of serotonin in the action of atypical antipsychotic drugs. Clin. Neurosci.

[36] Meltzer HY, Li Z, Kaneda Y, Ichikawa J (2003). Serotonin receptors: their key role in drugs to treat schizophrenia. Prog. Neuro-Psychopharmacol. Biol. Psychiatry.

[37] Baker RW, Chengappa KN, Baird JW, Steingard S, Christ MA, Schooler NR (1992). Emergence of obsessive compulsive symptoms during treatment with clozapine. J. Clin. Psychiatry.

[38] de Haan L, Linszen DH, Gorsira R (1999). Clozapine and obsessions in patients with recent-onset schizophrenia and other psychotic disorders. J. Clin. Psychiatry.

[39] Lykouras L, Alevizos B, Michalopoulou P, Rabavilas A (2003). Obsessive-compulsive symptoms induced by atypical antipsychotics. A review of the reported cases. Prog. Neuro-Psychopharmacol. Biol. Psychiatry.

[40] Kwon JS, Joo YH, Nam HJ, Lim M, Cho EY, Jung MH, Choi JS, Kim B, Kang DH, Oh S, Park T, Hong KS (2009). Association of the glutamate transporter gene SLC1A1 with atypical antipsychotics-induced obsessive-compulsive symptoms. Arch. Gen. Psychiatry.

[41] Reznik I, Yavin I, Stryjer R, Spivak B, Gonen N, Strous R, Mester R, Weizman A, Kotler M (2004). Clozapine in the treatment of obsessive-compulsive symptoms in schizophrenia patients: a case series study. Pharmacopsychiatry.

[42] de Haan L, Oekeneva A, van AT, Linszen D (2004). Obsessive-compulsive disorder and treatment with clozapine in 200 patients with recent-onset schizophrenia or related disorders. Eur Psychiatry: J. Assoc. Eur. Psychiatrists.

[43] Kang X, Simpson GM (2010). Clozapine: More side effects but still the best Antipsychotic. J. Psychiatry.

[44] Joober R, Boksa P (2010). Clozapine: a distinct, poorly understood and under-used molecule. J. Psychiatry Neurosci.

[45] Kane J, Honigfeld G, Singer J, Meltzer H (1988). Clozapine for the treatment-resistant schizophrenic. A double-blind comparison with chlorpromazine. Arch. Gen. Psychiatry.

[46] Asenjo L C, Komossa K, Rummel-Kluge C, Hunger H, Schmid F, Schwarz S, Leucht S (2010). Clozapine versus other atypical antipsychotics for schizophrenia. Cochrane Database Syst. Rev.

[47] McEvoy JP, Lieberman JA, Stroup TS, Davis SM, Meltzer HY, Rosenheck RA, Swartz MS, Perkins DO, Keefe RS, Davis CE, Severe J, Hsiao JK, Catie I, McEvoy JP, Lieberman JA, Stroup TS, Davis SM, Meltzer HY, Rosenheck RA, Swartz MS, Perkins DO, Keefe RSE, Davis CE, Severe J, Hsiao JK, Caite I (2006). Effectiveness of clozapine versus olanzapine, quetiapine, and risperidone in patients with chronic schizophrenia who did not respond to prior atypical antipsychotic treatment. Am. J. Psychiatry.

[48] Tiihonen J, Lonnqvist J, Wahlbeck K, Klaukka T, Niskanen L, Tanskanen A, Haukka J (2009). 11-year follow-up of mortality in patients with schizophrenia: a population-based cohort study (FIN11 study). Lancet.

[49] Fenton WS, McGlashan TH (1986). The prognostic significance of obsessive-compulsive symptoms in schizophrenia. Am. J. Psychiatry.

[50] Berman I, Kalinowski A, Berman SM, Lengua J, Green AI (1995). Obsessive and compulsive symptoms in chronic schizophrenia. Compr. Psychiatry.

[51] Berman I, Sapers BL, Chang HH, Losonczy MF, Schmildler J, Green AI (1995). Treatment of obsessive-compulsive symptoms in schizophrenic patients with clomipramine. J. Clin. Psychopharmacol.

[52] Nolfe G, Milano W, Zontini G, Petrella C, De RM, Rundle-Smith S, Nolfe G (2010). Obsessive-compulsive symptoms in schizophrenia: their relationship with clinical features and pharmacological treatment. J. Psychiatric Practice.

[53] Hippius H (1989). The history of clozapine. Psychopharmacology.

[54] Niendam T A, Berzak J, Cannon TD, Bearden CE (2009). Obsessive compulsive symptoms in the psychosis prodrome: Correlates of clinical and functional outcome. Schizophrenia Res.

[55] Rubino IA, Frank E, Croce NR, Pozzi D, Lanza di ST, Siracusano A (2009). A comparative study of axis I antecedents before age 18 of unipolar depression, bipolar disorder and schizophrenia. Psychopathology.

[56] Poyurovsky M, Fuchs C, Weizman A (1999). Obsessive-compulsive disorder in patients with first-episode schizophrenia. Am. J. Psychiatry.

[57] Sterk B, Lankreijer K, Linszen DH, de Haan L (2011). Obsessive-compulsive symptoms in first episode psychosis and in subjects at ultra high risk for developing psychosis; onset and relationship to psychotic symptoms. Austr. New Zealand J. Psychiatry.

[58] Shioiri T, Shinada K, Kuwabara H, Someya T (2007). Early prodromal symptoms and diagnoses before first psychotic episode in 219 inpatients with schizophrenia. Psychiatry Clin. Neurosci.

[59] Zink M, Knopf U, Kuwilsky A (2006). Management of clozapine-induced obsessive compulsive symptoms in a man with schizophrenia. Austr. New Zealand J. Psychiatry.

[60] Zink M, Englisch S, Knopf U, Kuwilsky A, Dressing H (2007). Augmentation of clozapine with valproic acid for clozapine-induced obsessive compulsive symptoms. Pharmacopsychiatry.

[61] Englisch S, Esslinger C, Inta D, Weinbrenner A, Peus V, Gutschalk A, Schirmbeck F, Zink M (2009). Clozapine-induced obsessive compulsive syndromes improve in combination with aripiprazole. Clin. Neuropharmacol.

[62] Schirmbeck F, Esslinger C, Rausch F, Englisch S, Meyer-Lindenberg A, Zink M (2011). Antiserotonergic antipsychotics are associated with obsessive-compulsive symptoms in schizophrenia. Psychol. Med.

[63] Lin SK, Su SF, Pan CH (2006). Higher plasma drug concentration in clozapine-treated schizophrenic patients with side effects of obsessive/compulsive symptoms. Ther. Drug Monit.

[64] Lim M, Park DY, Kwon JS, Joo YH, Hong KS (2007). Prevalence and clinical characteristics of obsessive-compulsive symptoms associated with atypical antipsychotics. J. Clin. Psychopharmacol.

[65] Ertugrul A, nil Yagcioglu AE, Eni N, Yazici KM (2005). Obsessive-compulsive symptoms in clozapine-treated schizophrenic patients. Psychiatry Clin. Neurosci.

[66] Shapiro DA, Renock S, Arrington E, Chiodo LA, Liu LX, Sibley DR, Roth BL, Mailman R (2003). Aripiprazole a novel atypical antipsychotic drug with a unique and robust pharmacology. Neuropsychopharmacology.

[67] Meltzer HY, Sumiyoshi T (2008). Does stimulation of 5-HT(1A) receptors improve cognition in schizophrenia?. Behav. Brain Res.

[68] Lopez-Gil X, Artigas F, Adell A (2010). Unraveling monoamine receptors involved in the action of typical and atypical antipsychotics on glutamatergic and serotonergic transmission in prefrontal cortex. Curr. Pharm. Design.

[69] Meltzer HY, Huang M (2008). *In vivo* actions of atypical antipsychotic drug on serotonergic and dopaminergic systems. Prog. Brain Res.

[70] Remington G (2008). Alterations of dopamine and serotonin transmission in schizophrenia. Prog. Brain Res.

[71] Englisch S, Zink M (2008). Combined antipsychotic treatment involving clozapine and aripiprazole. Prog. Neuro-Psychopharmacol. Biol. Psychiatr.

[72] Chang JS, Ahn Y-M, Park HJ, Lee KY, Kim SH, Kang UG, Kim JS (2008). Aripiprazole augmentation in clozapine-treated patients with refractory Schizophrenia: An 8-Week, randomized, double-blind, placebo-controlled trial. J. Clin. Psychiatry.

[73] Connor KM, Payne VM, Gadde KM, Zhang W, Davidson JR, Connor KM, Payne VM, Gadde KM, Zhang W, Davidson JRT (2005). The use of aripiprazole in obsessive-compulsive disorder: preliminary observations in 8 patients. J. Clin. Psychiatry.

[74] Kim SW, Shin IS, Kim JM, Yang SJ, Hwang MY, Yoon JS (2008). Amisulpride improves obsessive-compulsive symptoms in schizophrenia patients taking atypical antipsychotics: an open-label switch study. J. Clin. Psychopharmacol.

[75] Pani L, Villagran JM, Kontaxakis VP, Alptekin K (2008). Practical issues with amisulpride in the management of patients with schizophrenia. Clin. Drug Investig.

[76] de Haan L, Beuk N, Hoogenboom B, Dingemans P, Linszen D (2002). Obsessive-compulsive symptoms during treatment with olanzapine and risperidone: a prospective study of 113 patients with recent-onset schizophrenia or related disorders. J. Clin. Psychiatry.

[77] Rocha FL, Hara C (2006). Benefits of combining aripiprazole to clozapine: Three case reports. Prog. Neuro-Psychopharmacol. Biol. Psychiatry.

[78] Peters B, de HL (2009). Remission of schizophrenia psychosis and strong reduction of obsessive-compulsive disorder after adding clozapine to aripiprazole. Prog. Neuro-Psychopharmacol. Biol. Psychiatry.

[79] Bloch MH, Landeros-Weisenberger A, Kelmendi B, Coric V, Bracken MB, Leckman JF (2006). A systematic review: Antipsychotic augmentation with treatment refractory obsessive-compulsive disorder. Mol. Psychiatry.

[80] Bandelow B, Zohar J, Hollander E, Kasper S, ller HJ, Zohar J, Hollander E, Kasper S, ller H.J, Bandelow B, Allgulander C, yuso-Gutierrez J, Baldwin D.S, Buenvicius R, Cassano G, Fineberg N, Gabriels L, Hindmarch I, Kaiya H, Klein DF, Lader M, Lecrubier Y, Pine JP, Liebowitz MR, Lopez-Ibor JJ, Marazziti D, Miguel EC, Oh KS, Preter M, Rupprecht R, Sato M, Starcevic V, Stein DJ, van AM, Vega J (2008). World Federation of Societies of Biological Psychiatry (WFSBP) guidelines for the pharmacological treatment of anxiety, obsessive-compulsive and post-traumatic stress disorders - first revision. World J. Biol. Psychiatry.

[81] Dold M, Aigner M, Lanzenberger R, Kasper S (2011). Efficacy of antipsychotic augmentation therapy in treatment-resistant obsessive-compulsive disorder - A meta-analysis of double-blind, randomised, placebo-controlled trials. Fortschritte der Neurologie-Psychiatrie.

[82] Pogarell O, Hamann C, Poepperl G, Juckel G, Chouker M, Zaudig M, Riedel M, Moeller HJ, Hegerl U, Tatsch K (2003). Elevated brain serotonin transporter availability in patients with obsessive-compulsive disorder. Biol. Psychiatry.

[83] Steingard S, Chengappa KNR, Baker R, Schooler NR (1993). Clozapine, obsessive symptoms, and serotonergic mechanisms. Am. J. Psychiatry.

[84] Meltzer HY (1994). An overview of the mechanism of action of clozapine. J. Clin. Psychiatry.

[85] Coward DM (1992). General pharmacology of clozapine. Br. J. Psychiatry - Supplementum.

[86] Linden DE (2006). How psychotherapy changes the brain - the contribution of functional neuroimaging. Mol. Psychiatry.

[87] Saxena S, Gorbis E, O'Neill J, Baker SK, Mandelkern MA, Maidment KM, Chang S, Salamon N, Brody AL, Schwartz JM, London ED (2009). Rapid effects of brief intensive cognitive-behavioral therapy on brain glucose metabolism in obsessive-compulsive disorder. Mol. Psychiatry.

[88] Dickel DE, Veenstra-Vander WJ, Cox NJ, Wu X, Fischer DJ, Van, Etten-Lee M, Himle JA, Leventhal BL, Cook EH, Hanna GL (2006). Association testing of the positional and functional candidate gene SLC1A1/EAAC1 in early-onset obsessive-compulsive disorder. Arch. Gen. Psychiatry.

[89] Arnold PD, Sicard T, Burroughs E, Richter MA, Kennedy JL (2006). Glutamate transporter gene SLC1A1 associated with obsessive-compulsive disorder. Arch. Gen. Psychiatry.

[90] Veenstra-Vander WJ, Kim SJ, Gonen D, Hanna GL, Leventhal BL, Cook EH (2001). Genomic organization of the SLC1A1/EAAC1 gene and mutation screening in early-onset obsessive-compulsive disorder. Mol. Psychiatry.

[91] Schirmbeck F, Nieratschker V, Frank J, Englisch S, Rausch F, Meyer-Lindenberg A, Rietschel M, Zink M (2011). No evidence for associations of polymorphisms in the glutamate transporter gene *SLC1A1* and obsessive compulsive symptoms induced by second generation antipsychotic agents. Pharmacogenom. J.

[92] Berman I, Merson A, Viegner B, Losonczy MF, Pappas D, Green AI (1998). Obsessions and compulsions as a distinct cluster of symptoms in schizophrenia: A neuropsychological study. J. Nervous Mental Dis.

[93] Sliman EB (2007). Differentielle neurokognitive Defizite bei Zwangsstörungen in Ab-hängigkeit vom klinischen Störungsbild und dem Ausprägungsgrad der Symptomatik im Vergleich zu Patienten mit Schizophrenie und gesunden Kontrollen. Dissertation Ruhr University Bochum.

[94] Lysaker PH, Whitney KA, Davis LW (2009). Associations of executive function with concurrent and prospective reports of obsessive-compulsive symptoms in schizophrenia. J. Neuropsychiatry Clin. Neurosci.

[95] Lysaker PH, Bryson GJ, Marks KA, Greig TC, Bell MD (2002). Association of obsessions and compulsions in schizophrenia with neurocognition and negative symptoms. J. Neuropsychiatry Clin. Neurosci.

[96] Patel DD, Laws KR, Padhi A, Farrow JM, Mukhopadhaya K, Krishnaiah R, Fineberg NA (2010). The neuropsychology of the schizo-obsessive subtype of schizophrenia: a new analysis. Psychol. Med.

[97] Hwang MY, Kim SW, Yum SY, Opler LA (2009). Management of schizophrenia with obsessive-compulsive features. Psychiatric Clin. North Am.

[98] Poyurovsky M, Glick I, Koran LM (2010). Lamotrigine augmentation in schizophrenia and schizoaffective patients with obsessive-compulsive symptoms. J. Psychopharmacol.

